# RNA sequencing of the lncRNA regulome modulated by *Withania somnifera* in human neuroblastoma SK-N-SH cells

**DOI:** 10.1007/s11033-025-10981-7

**Published:** 2025-09-10

**Authors:** Eshita Sharma, Dilip Mehta, Simran Sahare, Saiprasad Ajgaonkar, Praful Saha, Anand Bhaskar, Jash Trivedi, Dhananya S., Manju Moorthy, Gopalakrishna Ramaswamy, Sujit Nair

**Affiliations:** 1 Phytoveda Pvt. Ltd, Mumbai, 400022 India; 2 Viridis Biopharma Pvt. Ltd, Mumbai, 400022 India; 3 Burnett School of Biomedical Sciences, University of Central Florida, Orlando, FL 32827 USA; 4 TheraCUES Innovations Pvt. Ltd, Bengaluru, 560092 India

**Keywords:** Long-chain noncoding RNAs, Human neuroblastoma cell line (SK-N-SH), RNA sequencing, Bioinformatics, Neurodegenerative diseases

## Abstract

**Background:**

The dysregulation of long-chain noncoding RNAs (lncRNAs) causes several complex human diseases including neurodegenerative disorders across the globe.

**Methods and results:**

This study aimed to investigate lncRNA expression profiles of *Withania somnifera* (WS)-treated human neuroblastoma SK-N-SH cells at different timepoints (3 & 9 h) and concentrations (50 & 100 µg/mL) using RNA sequencing. Differential gene expression analysis showed a total of 4772 differentially expressed lncRNAs, out of which 3971 were upregulated and 801 were downregulated compared to controls. Differential gene expression was observed in dose-dependent (30 upregulated, 25 downregulated, 100 µg/mL 3 h vs. 50 µg/mL 3 h; 36 upregulated, 247 downregulated, 100 µg/mL 9 h vs. 50 µg/mL 9 h) and temporal kinetics (79 upregulated, 64 downregulated, 50 µg/mL 9 h vs. 50 µg/mL 3 h; 22 upregulated, 200 downregulated, 100 µg/mL 9 h vs. 100 µg/mL 3 h). Enrichment analysis showed that modulated lncRNAs were mainly implicated in GPCR ligand binding, HDACs and HATs histones, cellular senescence, cell cycle and post-translational protein modifications. Dysregulated lncRNAs upon WS treatment included BACE1-AS, MALAT1, SNHG1, HOTAIR, MEG3, BDNF-AS, and SHANK2-AS1 which are potential biomarkers in several neurodegenerative diseases. Co-expression analysis revealed that genes such as HMOX1, CHGB, SLC7A11, NOS1, KCNJ and NPY2R may be important in neurodegenerative disorders.

**Conclusions:**

Taken together, our results indicated that WS treatment modulated several differentially expressed lncRNAs with putative regulatory potential in various neurodegenerative disorders. To the best of our knowledge, the lncRNA regulome that elicits the health-beneficial effects of WS has not been delineated thus far.

**Supplementary Information:**

The online version contains supplementary material available at 10.1007/s11033-025-10981-7.

## Introduction

Neurodegenerative diseases featuring neuronal cells loss include Alzheimer’s disease (AD), amyotrophic lateral sclerosis, Parkinson’s disease (PD), and Huntington’s disease [1]. At present, no treatments are available for such diseases which in turn pose a huge burden to society, families and individuals, thereby affecting the lifestyle as well as health of patients along with their caretakers. Evidence has revealed the involvement of numerous pathophysiological mechanisms in the pathogenesis of these neurodegenerative diseases including oxidative stress, mitochondrial dysfunction, inflammatory reactions and excitotoxic amino acids. However, the primary causes of neurodegenerative diseases are still unknown [2–4]. The poor diagnosis and difficulty in assessing such diseases call for novel biomarker development which can aid in early detection along with immediate treatment to prevent the progression of such diseases.

In addition to coding genes, noncoding RNAs such as microRNAs have been known to regulate human disease [5]. In recent times, long non-coding RNAs (lncRNAs) have emerged as novel genetic biomarkers and indispensable players in the diagnosis, development as well as therapeutics of neurodegenerative diseases [6, 7]. lncRNAs, composed of more than 200 nucleotides, are important biomolecules in cellular mechanisms participating in normal development and disease progression, playing a crucial role in disease regulation along with normal cellular processes *via* gene regulation [8]. For example, the lncRNA CHASERR has been implicated in glioma progression [9] and a lncRNA/circRNA-miRNA-mRNA network has been reported in nasopharyngeal cancer [10]. Moreover, lncRNA LINC00657 sponges miR-590-3p in the regulation of angiogenesis [11].

*Withania somnifera* (WS) or Ashwagandha, commonly recognized as the “Winter Cherry” or “Indian Ginseng” has been known for its therapeutic activities including antioxidant, antiinflammation, anticancer, neuroprotection and modulation of the immune system [12]. Additionally, WS modulates RNA-associated pathways which can entail transcriptional, post-transcriptional and epigenetic modifications in gene expression and signaling networks and can be exploited for treatment of various neurodegenerative disorders [13]. Apelin-13 has been reported to protect against neuroinflammation in AD [14], however, the role of WS in the regulation of lncRNAs in neurodegeneration has not been explored thus far.

RNA sequencing has been widely exploited to measure gene expression alterations, discovery of novel transcripts as well as their roles in diseased states. Identifying differentially expressed genes (DEGs), isoforms and prediction of novel genes as putative drug targets are emerging research areas [15]. Several investigations have highlighted the significance of lncRNAs in the etiology of neurodegenerative diseases [16–19]. In a microarray analysis of lncRNAs expressed in the hippocampal region in AD, 315 lncRNAs were found to be dysregulated [20]. In PD, 87 lncRNAs were reported to be significantly modulated in the substantia nigra in the human neuroblastoma SH-SY5Y cell line, out of which two crucial lncRNAs i.e., AL049437 and AK021630 were further knocked-out. Reduction in AL049437 expression caused enhanced cell viability, mitochondrial membrane potential, mass; and converse for AK021630 [21]. In AD, approximately 100 dysregulated lncRNA transcripts (40 up- and 60 downregulated) were reported in amyloid-treated SH-SY5Y cells [22]. EBF3-AS deficiency reduced EBF3 levels inhibiting okadaic acid and apoptosis triggered by Aβ treatment in SH-SY5Y cells [17]. Therefore, such studies highlight the crucial role of lncRNAs in pathogenesis of brain-related complexities. Hence, it may be important to investigate novel lncRNAs associated with neurodegenerative disorders.

There is a scarcity of literature on the effect of WS treatment on modulation of lncRNAs expression involved in brain-related complexities. In this study, high-throughput RNA-sequencing was undertaken to investigate the effect of WS treatment on alteration of lncRNA expression in human neuroblastoma SH-SY5Y cells at different concentrations and time-points. Further, differential gene expression (DGE) analysis and enrichment analysis as well as co-expression analysis of lncRNA-mRNA was also conducted. The key lncRNA biomarkers associated with neuroprotection of WS were explored. This will help to augment our understanding of the etiology of brain-related diseases as well as identify novel biomarkers in the lncRNA regulome which may act in concert with other transcriptional and/or non-transcriptional players to exert beneficial effects elicited by WS in neurodegenerative diseases. These lncRNAs may also serve as potential targets for future therapeutic intervention or as companion diagnostics for neurodegenerative diseases.

## Materials and methods

### Materials

Human neuroblastoma cell line (SK-N-SH) was acquired from the American Type Culture Collection (ATCC) (Rockville, MD, USA). Minimum Essential Medium Eagle (EMEM), fetal bovine serum (FBS), antibiotic solution exhibiting streptomycin (10,000 U/ml) and penicillin (10 mg/ml) were received from HiMedia, Mumbai, India. *Withania somnifera* (LongeFera™) was supplied by Phytoveda Pvt. Ltd., Mumbai, India. The powdered root extract of *Withania somnifera* comprised withaferin A, withanoside IV, withanoside V, 12-deoxy-withastramonolide, withanolide A, withanolide B and withanone as described earlier by Sharma et al.. (2025) [23]. 

### Cell culture and treatment

SK-N-SH cells were cultured in Minimum Essential Medium Eagle (EMEM) medium which was supplemented with 10% v/v FBS and 1% v/v Penicillin-Streptomycin incubated at 37 °C with 5% CO_2_ for 24 h. Following this, cells were seeded into 6-well plates and further incubated overnight in EMEM starvation medium containing 0.5% (v/v) FBS. Further, the cells were treated with *Withania somnifera* (LongeFera™) at two concentrations, i.e., 50 and 100 µg/mL for 3 and 9 h and control samples were treated with dimethyl sulfoxide (DMSO).

### RNA extraction, cDNA library preparation and sequencing

The extraction of total RNA from 12 samples was performed using the Total Aurum RNA Mini Kit (Bio-Rad, USA) as instructed in the manufacturer’s protocol. The concentrations and purity of RNA were determined utilizing a Qubit Fluorometer (Thermo Fisher Scientific, USA) with the Qubit RNA HS Assay Kit (Thermo Fisher Scientific, USA). RNA quality was assessed using an Agilent 2100 Bioanalyzer (Agilent Technologies, USA) with the RNA 6000 Nano Kit, and samples with RNA integrity number (RIN) scoring between 9.3 and 9.8 were used for further analysis.

RNA sequencing and subsequent bioinformatics analyses were performed at TheraCUES Innovations Pvt. Ltd., Bengaluru. For RNA sequencing, ribosomal RNA (rRNA) constituting approximately 95% of the total RNA, was depleted using Qiaseq Fast Select rRNA-HMR (Qiagen, USA) to enrich the remaining RNA. The preparation of cDNA libraries was achieved using the NEB Ultra II Directional RNA-Seq Library Prep Kit (New England Biolabs, USA) following the manufacturer’s instructions. After library preparation, the quantification of libraries was completed using the Qubit HS Assay (Invitrogen, USA) and further evaluated for size distribution using the Agilent Fragment Analyzer with the HS NGS Fragment Kit (1–6000 bp) (Agilent Technologies, USA). The pooling and dilution of cDNA libraries were performed to accomplish the optimal loading concentration. Sequencing was performed on an Illumina NovaSeq platform (Illumina, USA) which generates 150 bp paired end reads for each sample. The sequencing depth and quality were found to be sufficient for subsequent DGE and lncRNA analyses.

### RNA sequence data analysis

The quality of raw sequencing reads was evaluated using FastQC (v0.11.9) to assess base quality distributions, GC content, and potential adapter contamination. Low-quality sequences and adapter sequences were removed using Cutadapt (v3.5). Following trimming, the alignment of high-quality paired-end reads was performed to the GRCh38 human reference genome *via* the splice-aware aligner STAR (v2.7.11a), ensuring accurate mapping of reads to the genome, including exon-exon junctions. Alignment metrics, including the percentage of uniquely mapped reads were checked for each sample. Gene expression was quantified using Feature Counts (v2.0.1) against the Gencode v44 GTF annotation, producing raw read counts for each gene, including both protein-coding and noncoding transcripts.

###  Differential LncRNA expression analysis

Differential expression of both known and novel lncRNAs was performed using DESeq2 in the R program with raw read counts normalized to account for library size and composition. Genes with a log2 fold change ≥ 2 or ≤−2 along with p-value ≤ 0.05 were taken as differentially expressed. DEG analysis was performed for treated and untreated samples across various conditions. Treated samples included S_50_3hr, S_50_9hr, S_100_3hr, and S_100_9hr, while untreated samples included C_3hr, C_9hr, C_3hr, and C_9hr. DEG comparisons were performed between treated and untreated samples at corresponding timepoints and doses including S_50_3hr vs. C_3hr, S_50_9hr vs. C_9hr, S_100_3hr vs. C_3hr, and S_100_9hr vs. C_9hr. Additionally, DEG analysis was carried out to investigate changes over time within treated samples: S_50_9hr vs. S_50_3hr, and S_100_9hr vs. S_100_3hr. Finally, DEG analysis were used to examine the impact of different doses at the same timepoints: S_50_9hr vs. S_100_9hr and S_50_3hr vs. S_100_3hr.

### Functional enrichment analysis

Over representation analysis (ORA) for REACTOME pathways was performed separately for Positively and Negatively correlated mRNA targets (upregulated and downregulated mRNA targets) using DAVID webserver. Functional enrichment analysis of DEGs was conducted using ClusterProfiler, focusing on REACTOME pathways.

### Identification of known and novel LncRNAs

For the identification of known lncRNAs, the aligned reads were quantified using the Gencode lncRNA annotation file (gencode.v45.long_noncoding_RNAs.gtf) using FeatureCounts (2.0.1). Read count data were normalized using DESeq2. To identify novel lncRNAs, the mapped reads of each sample were reassembled and merged with StringTie. Further, known lncRNAs were identified by creating a combined database of lncRNAs from NONCODE, lncRNAKB, Gencode and searched against the re-assembled transcripts using CuffCompare, to obtain novel lncRNA transcripts. For lncRNA prediction, 3 types of transcripts (I, u, and x) were used, where I is transfrags falling entirely within a reference intron, u includes unknown and intergenic transcripts, and x is exonic overlap on the opposite strand with reference. Transcripts with length (≥ 200 bp), the number of exons (> 2), and open reading frame (ORF) length (< 300 bp) were taken. Coding potential of the identified transcripts was assessed using CPAT and PLEK. After removal of known lncRNA sequences, transcripts with coding potential scores less than 0 were assigned as potential novel lncRNAs. Presence of novel lncRNAs was checked using the parameters viz. transcripts with a length ≥ 200 bp, exons number > 2 and ORF < 300 followed by reassembling using StringTie. Only two novel transcripts satisfying the above conditions were detected.

### lncRNA-mRNA co-expression and network analysis

The top 10 hub lncRNAs (those with the highest number of mRNA targets) and their interaction with mRNAs were utilized for the lncRNA-mRNA co-expression network generation using Cytoscape 3.10.3. Co-expression of lncRNAs with mRNAs was analysed using Pearson correlation ≥ abs (0.9), pairs having R² ≥ 0.7, p-value ≤ 0.05 and target mRNA foldchange cutoff (of those top 10 lncRNA) > abs (log2Foldchange 3). Additionally, the classification of mRNA targets of lncRNAs into ‘cis’ & ‘trans’ based on co-localization was identified using FEElnc standalone software.

###  Statistical analyses

The analysis of lncRNA-seq was performed using DESeq2 in R (v4.2.1) program. Comparisons were made between untreated and treated groups at different time points and doses. Co-expression of lncRNAs and mRNAs was assessed using Pearson’s correlation, with significant correlations defined by R² ≥ 0.7 and p-value ≤ 0.05. Functional enrichment of significant DEGs was determined on the basis of adjusted p-value ≤ 0.05. The Benjamini-Hochberg correction (False Discovery Rate < 5%) was used.

### Quantitative Real-Time polymerase chain reaction (qRT-PCR) analyses

To confirm the RNA-Seq results, the expression levels of selected lncRNAs were measured using qRT-PCR. First-strand cDNA was synthesized from 4 µg of DNase I-treated total RNA using the QuantiTect Reverse Transcription Kit (Qiagen, USA). Primers specific to the lncRNAs were designed using the PrimerQuest Tool (Integrated DNA Technologies, IDT, Iowa, USA) and synthesized by IDT, Singapore (Table 1). β-actin was used as the internal control. qRT-PCR was performed in a 384-well format on the QuantStudio real-time PCR system (Applied Biosystems, USA), using SYBR Green Master Mix (Thermo Scientific, USA). The thermal cycling conditions were as follows: initial denaturation at 95 °C for 10 min, followed by 50 cycles of 95 °C for 15 s, 55 °C for 30 s, and 72 °C for 30 s. A dissociation curve analysis was conducted to ensure specificity of the amplification and to check for primer-dimer formation.

**Table 1:** List of primer sequences of select LncRNAs used for quantitative reverse transcriptase-polymerase chain reaction (qRT-PCR) analyses

S. no	Gene name	Gene symbol	Accession ID		Primer sequence
1	BACE1 antisense RNA	BACE1-AS	ENSG00000278768	Forward	5′-GGCACCTCCTAAGTGTACCTGC-3′
				Reverse	5′-CTCTCTGCTGGGCACGATTC-3′
2	Metastasis associated lung adenocarcinoma transcript 1	MALAT1	ENSG00000251562	Forward	5’-AAAGCAAGGTCTCCCCACAAG-3’
				Reverse	5’-GGTCTGTGCTAGATCAAAAGGCA-3’
3	HOX transcript antisense RNA	HOTAIR	ENSG00000228630	Forward	5’-CAGTGGGGAACTCTGACTCG-3’
				Reverse	5’-GTGCCTGGTGCTCTCTTACC-3’
4	Small nucleolar RNA host gene 1	SNHG1	ENSG00000255717	Forward	5’-ACGTTGGAACCGAAGAGAGC-3’
				Reverse	5’-GCAGCTGAATTCCCCAGGAT-3’
5	Maternally expressed gene 3	MEG3	ENSG00000214548	Forward	5’-GAATATGAGTTGTAAGTGGTAGAGTTT-3’
				Reverse	5’-TACAAACTTAACAAAAAAAAATCATACT-3’
6	Brain-derived neurotrophic factor antisense	BDNF-AS	ENSG00000245573	Forward	5’-CCGTGAGAAGATCTCATTGGGC-3’
				Reverse	5’-TGGGTCACAAGTCACGTAGC-3’
7	Growth arrest specific 5	GAS5	ENSG00000234741	Forward	5’-CTTCTGGGCTCAAGTGATCCT-3’
				Reverse	5’-TTGTGCCATGAGACTCCATCAG-3’
8	KCNQ1 opposite strand/antisense transcript 1	KCNQ1OT1	ENSG00000269821	Forward	5’-TTGGTAGGATTTTGTTGAGG-3’
				Reverse	5’-CAACCTTCCCCTACTACC-3’
9	Nuclear paraspeckle assembly transcript 1	NEAT1	ENSG00000245532	Forward	5’-GTAATTTTCGCTCGGCCTGG-3’
				Reverse	5’-TACCCGAGACTACTTCCCCA-3’
10	SH3 and multiple ankyrin repeat domains 2 antisense 1	SHANK2-AS1	ENSG00000226627	Forward	5’-GTGTTTCCGTAGAGTTGGGATAG-3’
				Reverse	5’- CAGTGTAATACCTTCGAGGTTCC-3’
11	β-actin	ACTB	PQ040393.1	Forward	5’-CCAACTGGGACGACATGGA-3’
				Reverse	5’-AGCCACACGCAGCTCATTG-3’

## Results

### PCA analysis and heatmap analysis for LncRNAs

**Fig. 1:**
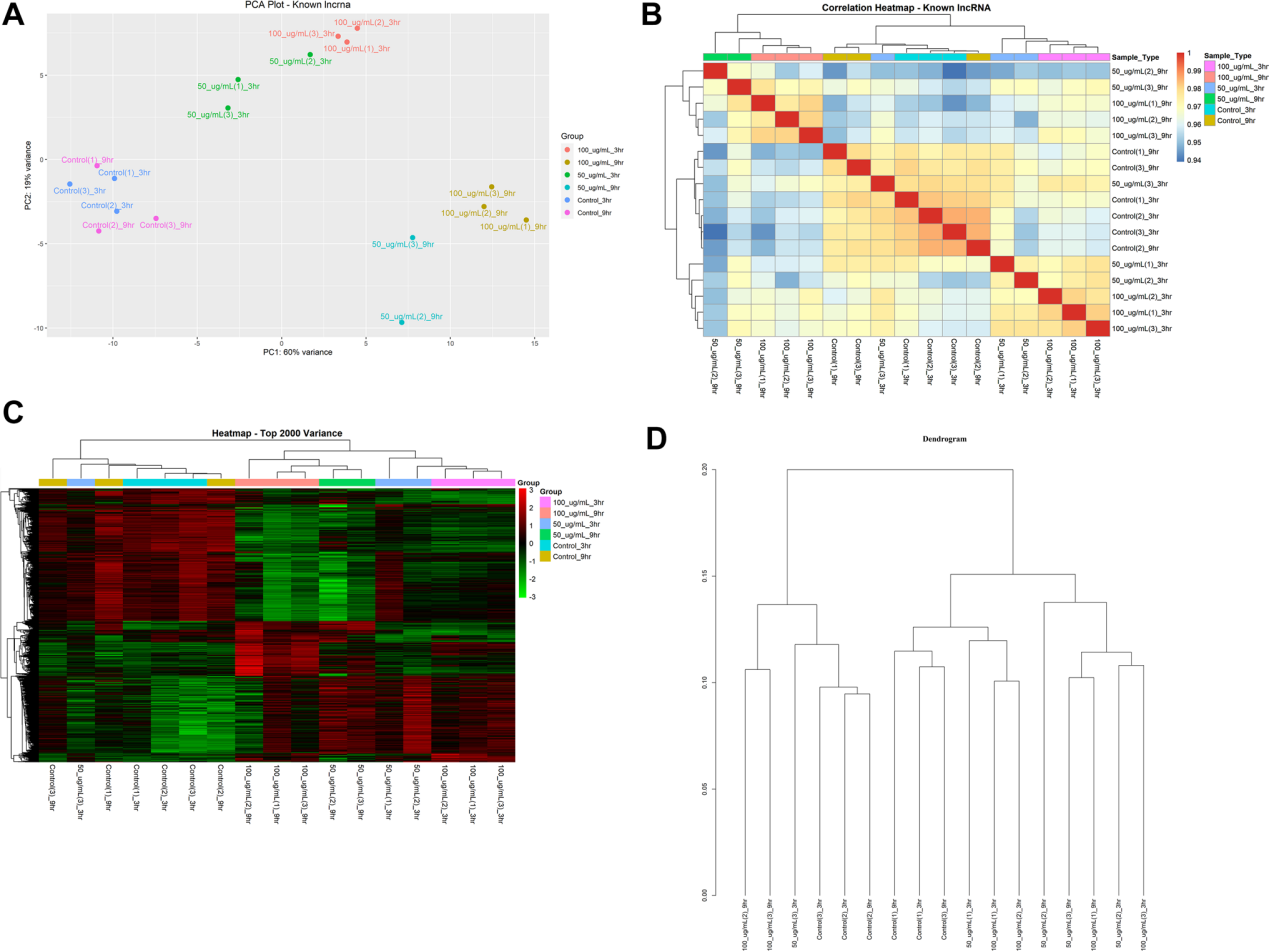
WS-treated human neuroblastoma SK-N-SH cells at various treatments. (**A**) Principal component analysis (PCA) plot of WS-treated human neuroblastoma SK-N-SH cells at different time points and dose concentrations; (**B**) Heatmap of top variable 2000 genes; (**C**) Sample-to-sample correlation; (**D**) Sample clustering dendrogram

The PCA plot in Fig. 1A is a principal component analysis plot of the treated and untreated samples in concentration and time-dependent approaches. Untreated samples C_3hr, C_9hr, C_3hr, and C_9hr were clustered together, while treated samples S_50_3hr, S_50_9hr, S_100_3hr, and S_100_9hr were clearly separated from the untreated samples. Additionally, samples treated for 9 h viz. S_50_9hr and S_100_9hr were separately clustered from those treated for 3 h i.e., S_50_3hr and S_100_3hr, indicating time-dependent changes in lncRNA expression.

Heatmap analysis revealed distinct clustering of untreated and treated samples separated by dose and time (Fig. 1B & C). Similarly, the dendrogram supported these findings, revealing a clear separation of untreated and treated samples, with treated samples further clustered based on concentration and time (Fig. 1D). These findings suggested that dose and duration of treatments may have a dynamic impact on gene expression profiles to identify possible treatment-responsive lncRNAs.

### Differential gene expression analysis

**Fig. 2:**
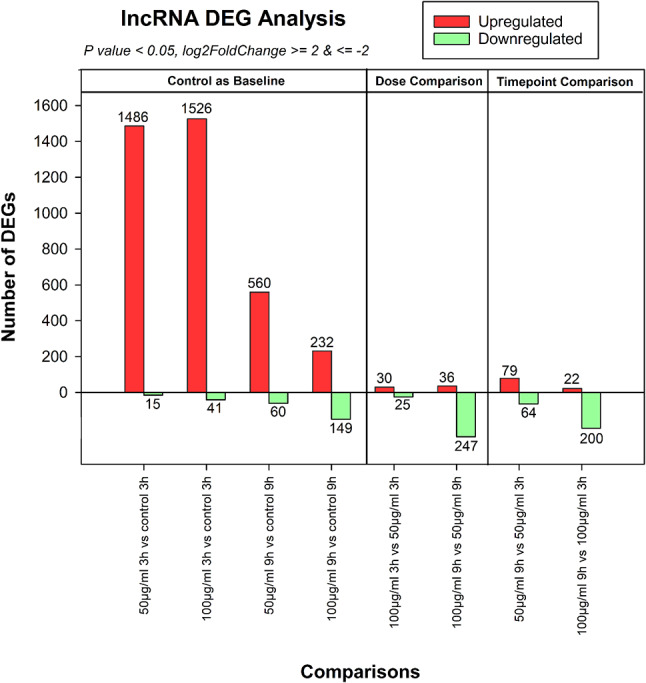
Differential gene expression analysis of lncRNAs at different time points and dose concentrations. Red and green colors indicate up or downregulation, respectively, of lncRNAs at statistical significance log2 fold change ≥ 2 and ≤ 2,* p*-value ≤ 0.05

The total number of lncRNAs tested was 20,052. DGE analysis revealed that 4772 differentially expressed lncRNAs were assembled using RNA sequencing; out of these, 3971 lncRNAs were found to be upregulated and 801 lncRNAs were downregulated when compared between untreated and treated groups (Fig. 2). In S_50_3hr vs. C_3hr, 1486 lncRNAs were reported as upregulated and 15 as downregulated. Similarly, 1526 upregulated and 41 downregulated lncRNAs were reported in S_100_3hr vs. C_3hr. Further, in S_50_9hr vs. C_9hr treatments, 560 lncRNAs were upregulated and 60 were downregulated, whereas 232 upregulated and 149 downregulated lncRNAs were observed in S_100_9hr vs. C_9hr (Fig. 2). Further, DGE analysis for dose comparisons revealed that 30 DEGs were upregulated and 25 were downregulated in S_100_3hr vs. S_50_3hr, while 36 DEGs were upregulated and 247 were downregulated in S_100_9hr vs. S_50_9hr (Fig. 2). Temporal comparisons demonstrated the upregulation of 79 DEGs and downregulation of 64 DEGs in S_50_9hr vs. S_50_3hr treatments; whereas 22 lncRNAs were found upregulated and 200 were reported downregulated in S_100_9hr vs. S_100_3hr (Fig. 2).

**Fig. 3:**
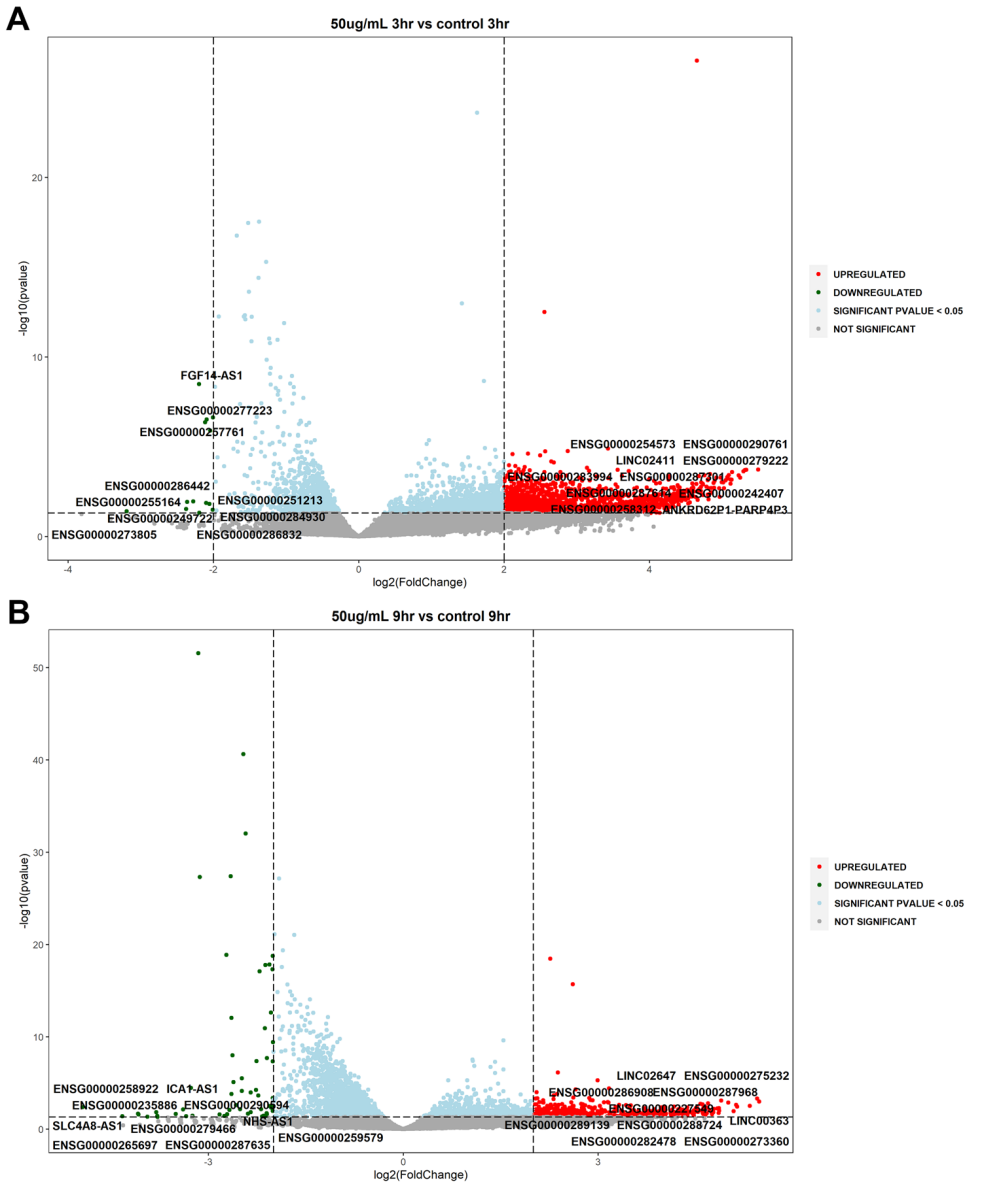

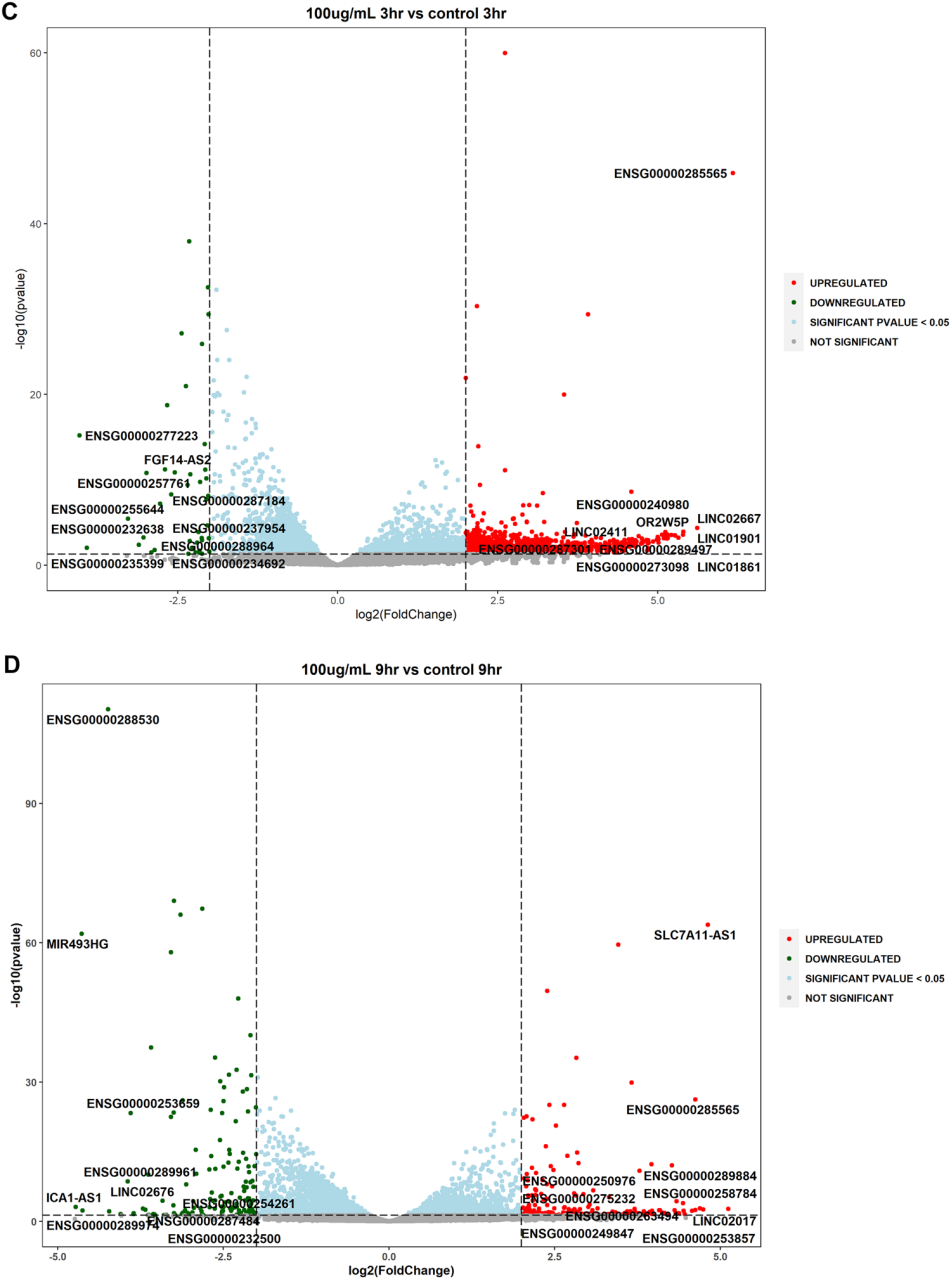

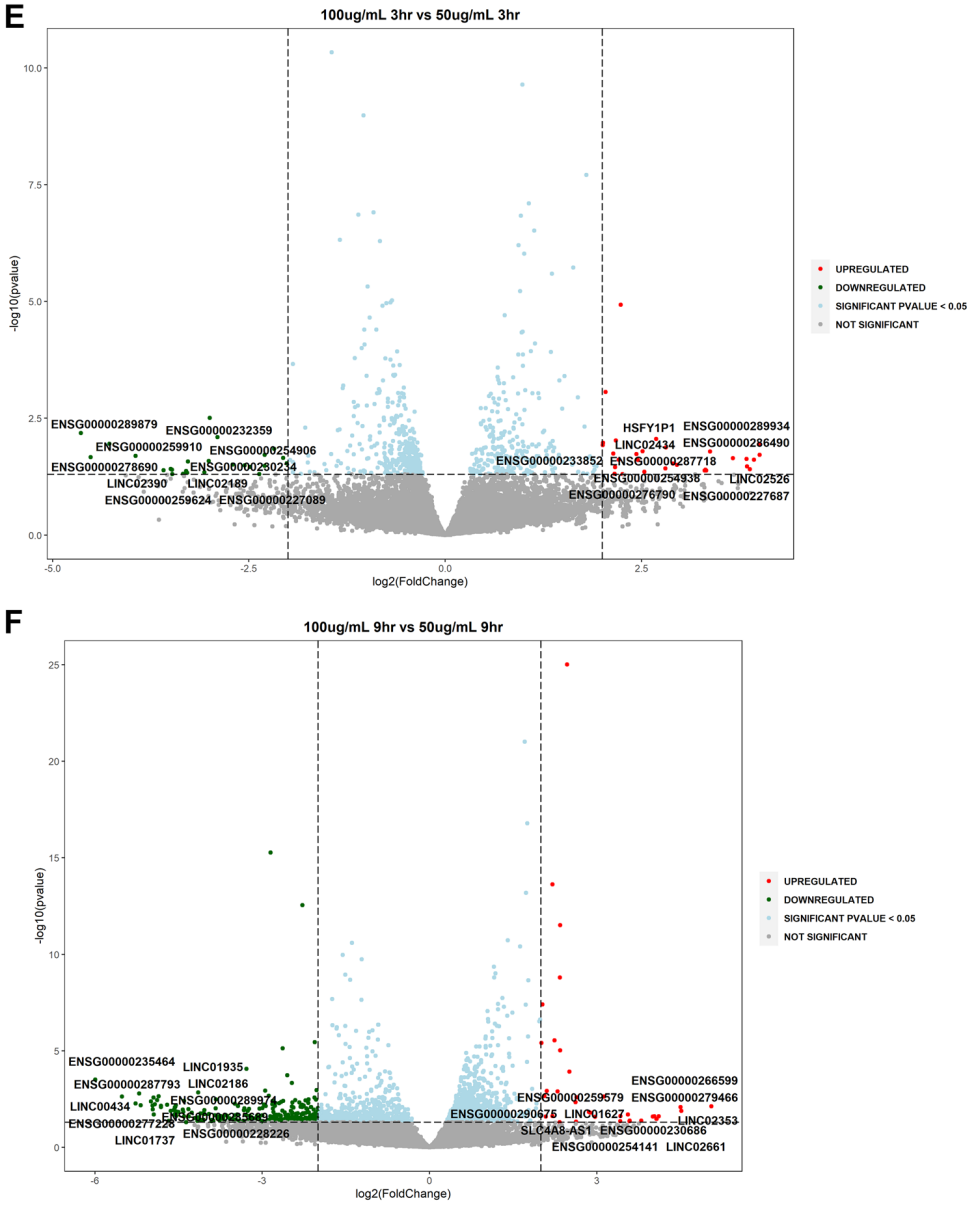

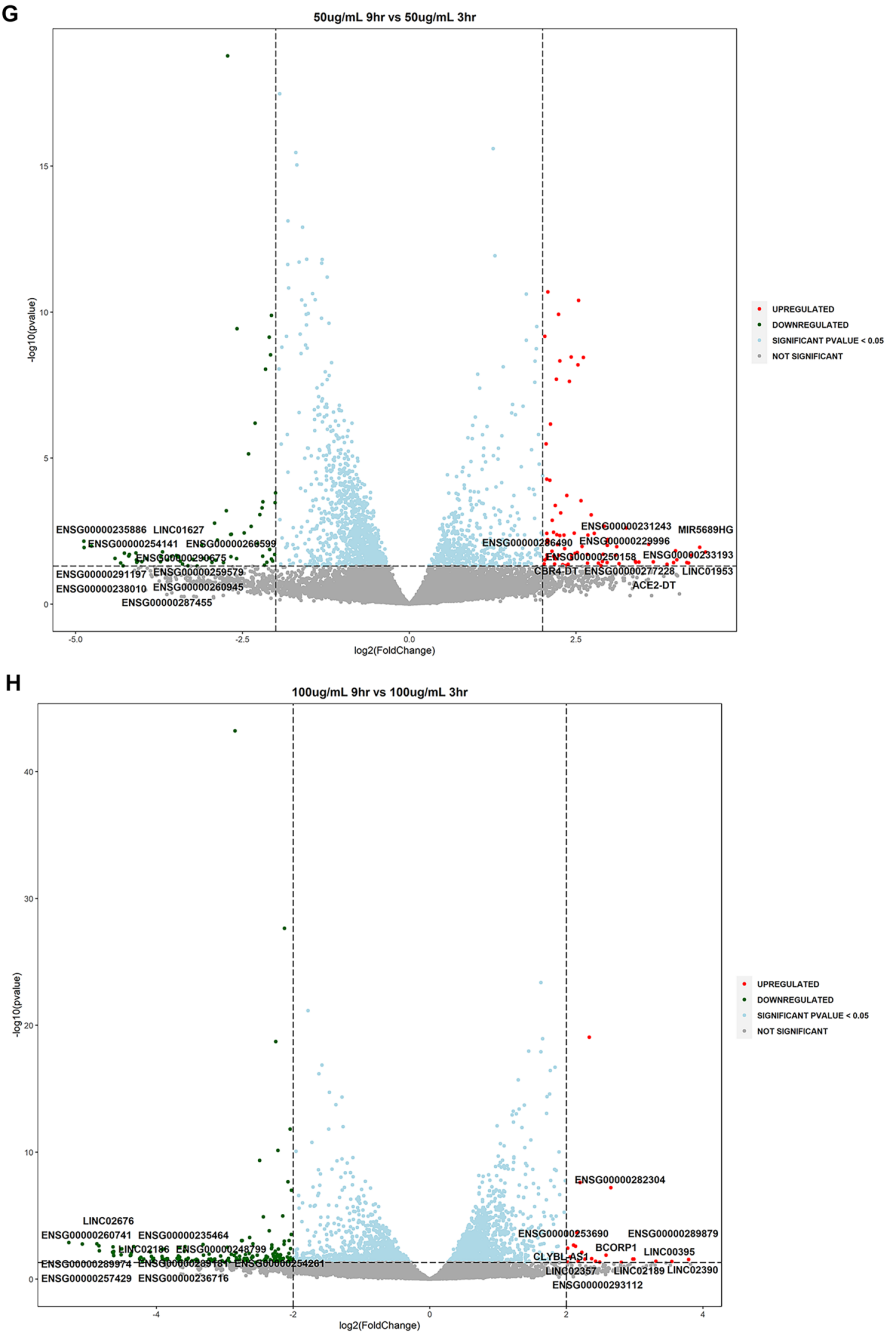
Volcano plots of differentially expressed lncRNAs in WS-treated SK-N-SH cells (**A**): S_50_3hr vs. C_3hr; (**B**): S_50_9hr vs. C_9hr; (**C**): S_100_3hr vs. S_100_3hr; (**D**): S_100_9hr vs. S_100_9hr; (**E**): S_100_3hr vs. S_50_3hr; (**F**): S_100_9hr vs. S_50_9hr; (**G**): S_50_9hr vs. S_50_3hr; (**H**): S_100_9hr vs. S_100_3hr. Volcano plots were prepared for differential gene expression analysis using log2 fold change as the abscissa and -log10 (p value) as the ordinate. The abscissa shows the differential expression fold change of the gene in different samples; the ordinate signifies the statistical significance of the difference in gene expression change; red color signifies the upregulated significant DEGs, green color denotes the downregulated significant DEGs; **DEGs**: differentially expressed genes

Volcano plots were utilized for the depiction of statistically significant lncRNAs by evaluating the differences in expressions between the untreated and treated samples along with dose and temporal comparisons (log2 fold change ≥ 2 or ≤ −2, *P* value ≤ 0.05). The analysis highlighted that several lncRNAs were upregulated and downregulated at statistical significance and are involved in various cellular processes such as oxidative stress, inflammation and neuronal function (Fig. 3A–H). Two novel lncRNAs were detected in this study viz., XLOC_023924 and XLOC_030213.

### REACTOME pathways enrichment analysis

REACTOME pathways enrichment analysis across various experimental conditions revealed significant alterations in cellular pathways. In S_50_3hr vs. C_3hr, upregulated mRNA targets included G-protein-coupled receptors (GPCR) ligand binding, GPCR signaling along with transport of salts, organic acids and amine compounds (Figure S1). Although REACTOME Pathway enrichment of S_50_9hr vs. C_9hr putatively demonstrated downregulation of DNA damage, cellular senescence, telomere stress-induced senescence and DNA methylation along with upregulation of pathways associated with GPCR ligand binding, peptide ligand-binding receptors, amine ligand-binding receptors, and GPCFR downstream signaling (Figure S2), it must be noted that there were only two replicates for S_50_9hr due to the removal of an outlier during PCA analysis, hence the results of this particular group may not be entirely reliable. Furthermore, enrichment analysis in S_50_9hr vs. C_9hr treatments showed putative upregulation of biological oxidations and peptide ligand-binding receptors (Figure S3), however, since this relates to the same S_50_9hr group as above, hence, the results for this group may not be completely relied upon. In S_100_9hr vs. C_9hr treatments, top 20 upregulated mRNA targets demonstrated significant enrichment in pathways associated with cellular response to stimuli and stress along with NFEL2L regulating antioxidant system upregulation, regulation of HSF1-mediated heat shock response, KEAP1-NFEL2L pathway, and HSF1-dependent transactivation (Figure S4). Conversely, the downregulated mRNA targets in similar comparison prominently featured the “HDACs deacetylate histones DNA methylation and HATs acetylate histones” pathways (Figure S4). Furthermore, in the temporal comparison, the downregulated mRNA targets showed significant enrichment in pathways related to histone modifications, chromatin organization and modifying enzymes (Figures S5, 6). In S_100_9hr vs. S_50_9hr, upregulated mRNA targets demonstrated significant enrichment in pathways related to cellular response to stimuli, starvation and heat stress, enhanced amino acid transport across the plasma membrane, tryptophan catabolism, transport of inorganic cations/oligopeptides, activation of genes in response to ER stress and PERK regulated gene expression (Figure S7). These findings indicated that both the concentration and duration of treatment significantly affect key cellular processes, particularly those involving stress responses and histone modifications.

### LncRNA and mRNA Co-expression network

**Fig. 4:**
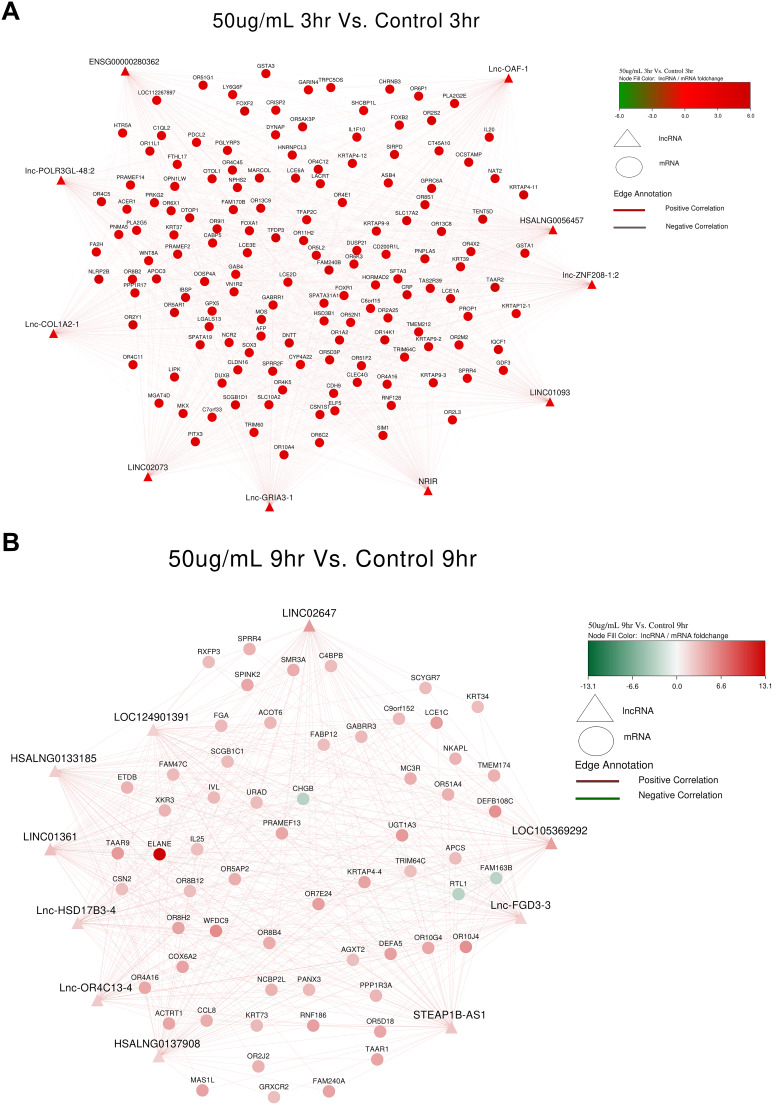

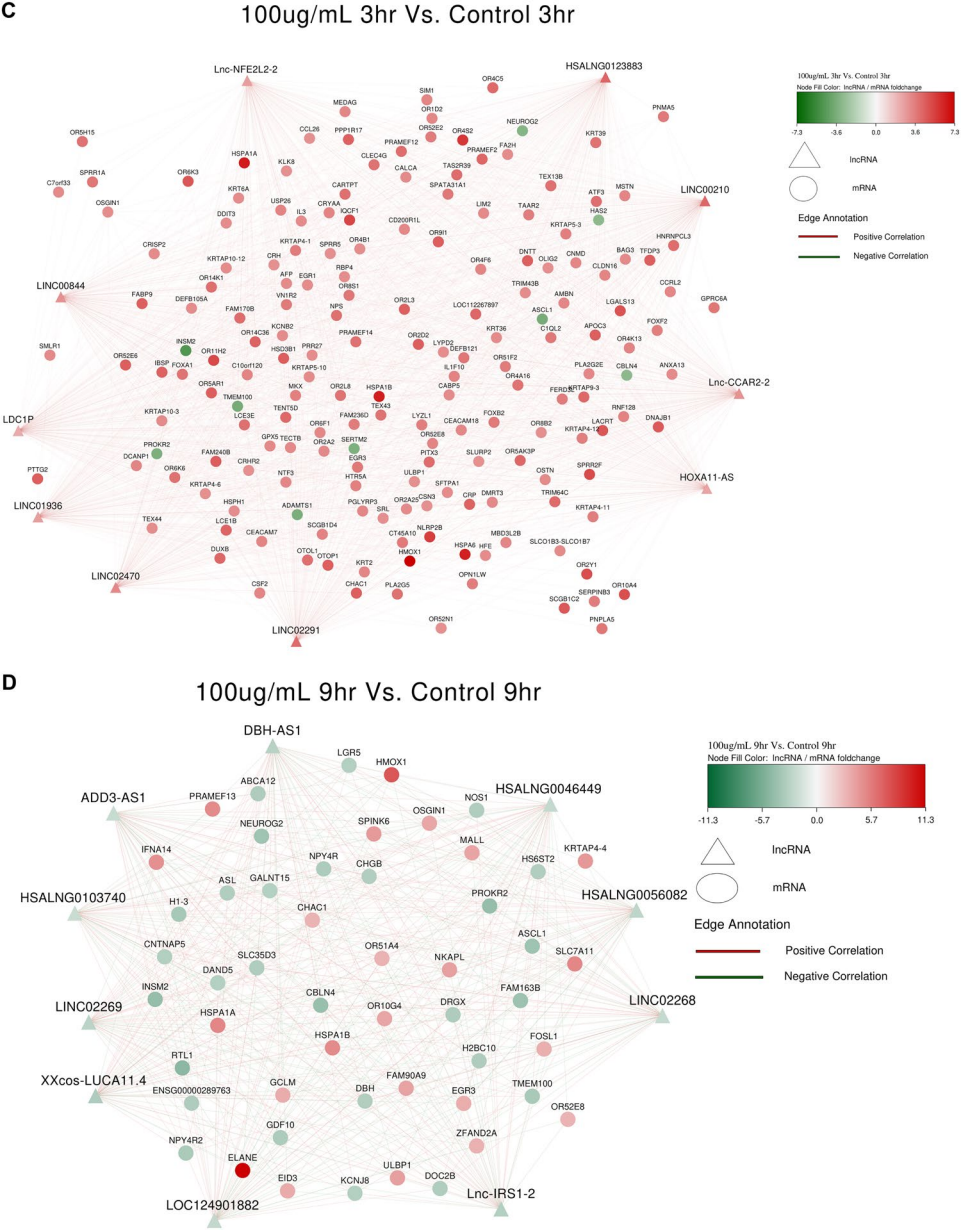
lncRNA-mRNA coexpression analysis based on Pearson’s correlation coefficient (**A**): S_50_3hr vs. C_3hr; (**B**): S_50_9hr vs. C_9hr; (**C**): S_100_3hr vs. S_100_3hr; (**D**): S_100_9hr vs. S_100_9hr. Red colour indicates upregulated mRNAs or lncRNAs; green color represents downregulated mRNAs or lncRNAs

A co-expression network of differentially expressed lncRNAs and significant mRNAs was constructed using Cytoscape version 3.10.3. In S_50_3hr vs. C_3hr, lnc-ZNF208-1:2, LINC02073, NRIR (negative regulator of interferon response) and HSALNG0056457 were upregulated (Fig. 4A). lnc-FGD3-3 (FYVE, RhoGEF and PH Domain-Containing Protein 3), STEAP1B-AS1 (transporter or ion channel) and LINC01361 (Long Intergenic Non-Protein Coding RNA) in S_50_9hr vs. C_9hr were putatively upregulated (Fig. 4B); however, given that there were only 2 samples in S_50_9hr after outlier removal in PCA analysis, the results of this group may be treated with caution. HOXA11-AS (homeobox A11 gene family), lnc-CCAR2-2 (Cell Cycle and Apoptosis Regulator 2), lnc-NFE2L2-2 (Basic leucine zipper (bZIP) proteins family) and LDC1P (Leucine Decarboxylase 1) in S_100_3hr vs. C_3hr were upregulated (Fig. 4C). lnc-IRS1-2 (IRS family proteins) and DBH-AS1 (DBH Antisense RNA 1) were found to be downregulated in S_100_9hr vs. C_9hr (Fig. 4D).

**Fig. 5:**
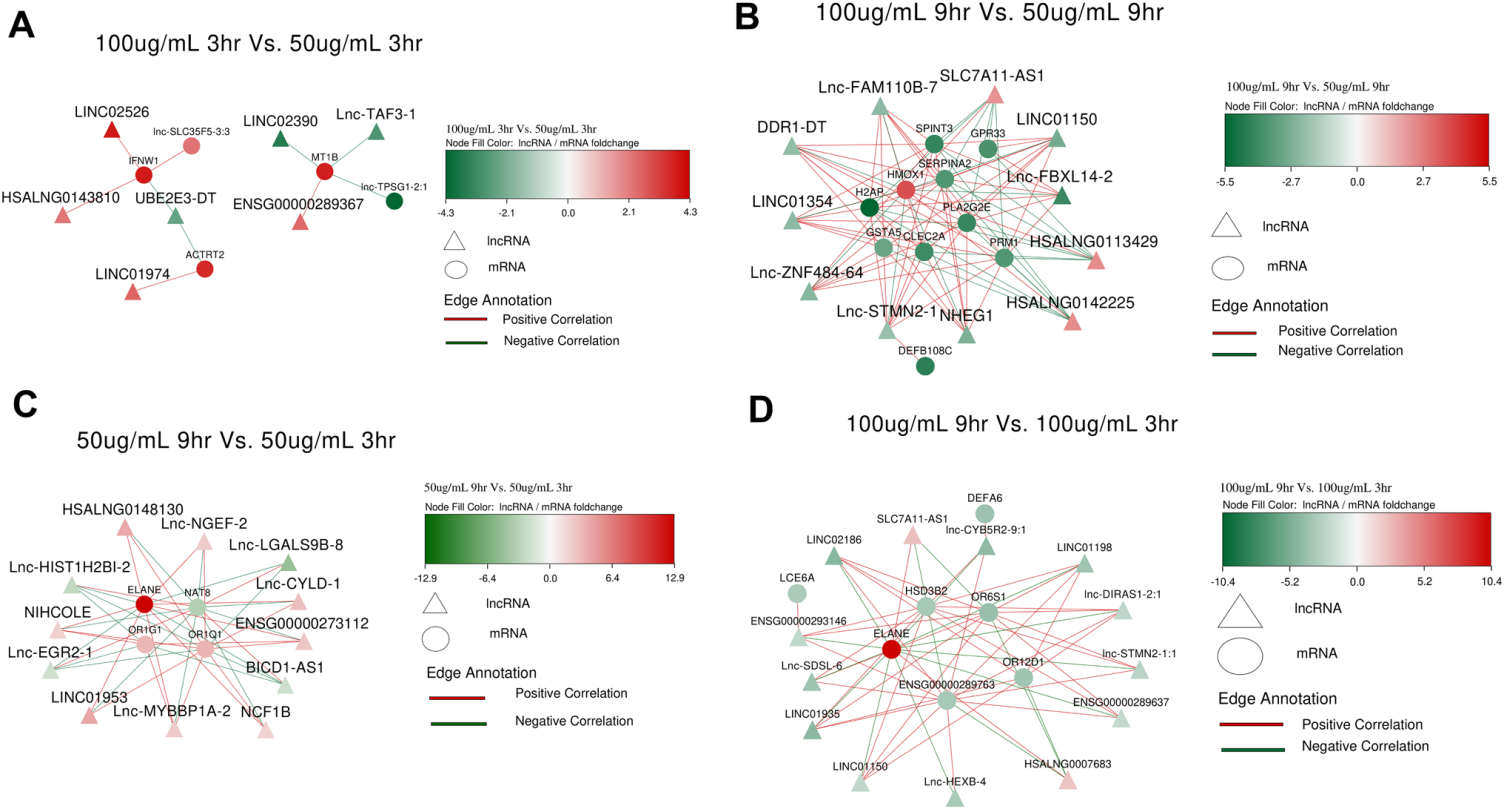
lncRNA-mRNA coexpression analysis based on Pearson’s correlation coefficient (**A**): S_100_3hr vs. C_3hr; (**B**): S_100_9hr vs. C_9hr; (**C**): S_50_9hr vs. C_3hr; (**D**): S_100_9hr vs. S_100_3hr. Red color indicates upregulated mRNAs or lncRNAs; green color represents downregulated mRNAs or lncRNAs

Dose and temporal comparison: In S_100_3hr vs. S_50_3hr, lnc-TAF3-1 (TATA-Box Binding Protein Associated Factor 3) was negatively correlated with MT1B, whereas ENSG00000289367 (Novel transcript) was positively correlated with MT1B; whereas UBE2E3-DT (ubiquitin conjugating enzyme E2 E3) was negatively correlated with IFNW1 (Fig. 5A). SLC7A11-AS1 (overlapping cis-natural anti-sense transcript) was upregulated whereas lnc-FAM110B-7 (Family With Sequence Similarity 110 Member B), lnc-ZNF484-64 (Zinc finger protein 484), lnc-STMN2-1 (TDP43 regulated gene), DDR1-DT (Discoidin Domain Receptor Tyrosine Kinase 1) and lnc-FBXL14-2 (Novel interaction partner of CDCP1) in S_100_9hr vs. S_50_9hr were downregulated (Fig. 5B). lnc-LGALS9B-8 (Galectin 9B) and lnc-EGR2-1 (transcription factor) were downregulated, whereas lnc-NGEF-2 (A novel member of the Dbl family of genes), lnc-CYLD-1 (Ubiquitin-specific protease (USP) cysteine proteases), NCF1B (Neutrophil Cytosolic Factor 1B (Pseudogene)), and lnc-MYBBP1A-2 (The nucleolar protein Myb-binding protein 1 A) were found to be upregulated in S_50_9hr vs. S_50_3hr (Fig. 5C). lnc-HEXB-4 (Hexosaminidase Subunit Beta), lnc-CYB5R2-9:1 (Cytochrome b5 reductase 2) and lnc-SDSL-6 (Serine Dehydratase Like) in S_100_9hr vs. S_100_3hr (Fig. 5D) were downregulated whereas SLC7A11-AS1 was upregulated.

### Quantitative Real-Time PCR expression analyses

**Fig. 6:**
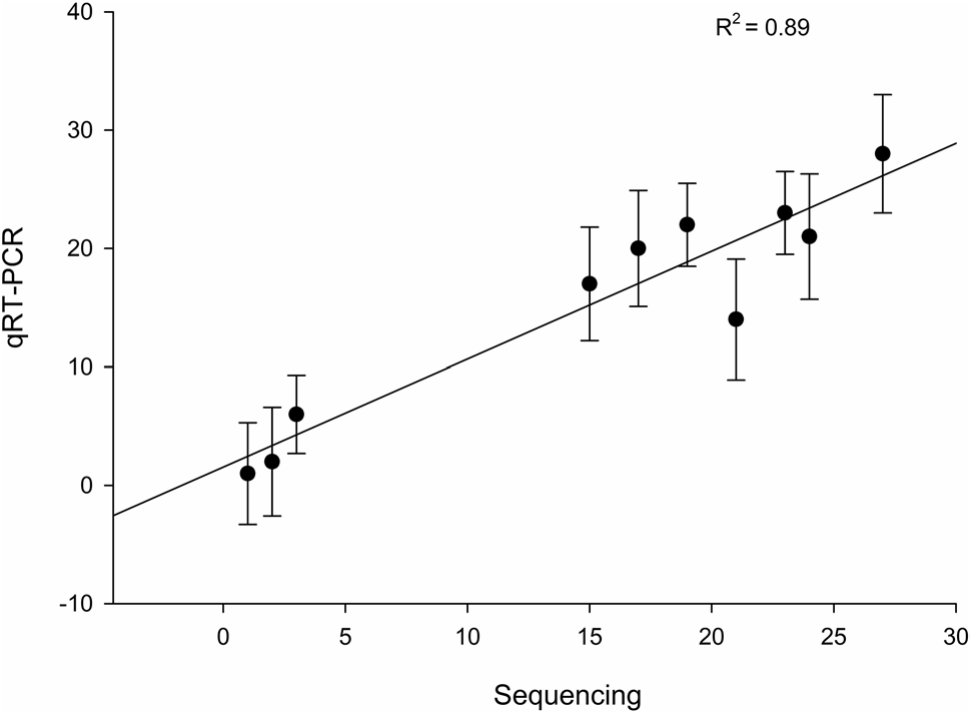
Validation of lncRNA-sequencing data for brain-related disorders. The values for fold change in gene expression by qRT-PCR were plotted against corresponding values in sequencing data (Spearman’s coefficient of determination, r^2 ^= 0.89)

To assess the consistency between qRT-PCR and RNA-seq, the expression patterns of selected lncRNAs were compared. A strong correlation was observed between the two methods, with a coefficient of determination (r²) of 0.89, indicating good agreement between the RNA-seq and qRT-PCR results (Fig. 6).

## Discussion

lncRNAs play a crucial role in gene regulation by mediating multiple pathways. Extensive research on neurodegenerative diseases has proven that differentially expressed lncRNAs were linked to the occurrence and development of neurodegenerative diseases [22, 24, 25]. WS is well known for its health attributes including neuroprotection against various brain-associated complexities [12, 13]. However, studies regarding the roles of lncRNAs and modulation by WS treatment in neuroblastoma cells is scarce in the public domain. Hence, this research was conducted to explore the effect of WS treatment on modulation of lncRNAs in human SK-N-SH cells to understand the key lncRNAs involved particularly in brain-associated diseases *via* RNA sequencing. The temporal or time-dependent expression of lncRNAs at 3h and 9h represents a time-dependent disruption of the homeostatic environment in the cellular milieu upon treatment with *Withania*, which is probably due to a temporal regulation of lncRNAs by *Withania.*

lncRNAs act as epigenetic regulators, affect chromatin modelling as well as histone modification, modulate translation *via* forming RNA duplex with mRNA, miRNA and protein sponges, thereby affecting mRNA expression at transcriptional and post-transcriptional levels. Based on this, co-expression analysis of mRNA-lncRNA was performed with filtered parameters. Additionally, mRNA classification targets of lncRNAs into ‘cis’ and ‘trans’ based on co-localization was identified in the current study. On the other hand, miRNAs situated in the intergenic regions of the genome possess their own independent cis-regulatory elements. miRNAs regulate mRNA expression post- transcriptionally by binding to complementary sequences in the untranslated regions of the target mRNAs. Thus, mRNAs and lncRNAs act potentially in concert via modulation of some miRNAs.

A single lncRNA may target multiple transcripts or multiple lncRNAs may be regulated by specific transcripts causing lncRNA-mediated transformations in various cellular pathways. In our study, we observed the downregulation of HDACs, HATs and chromatin organization which further control the cellular processes including chromatin modification, DNA replication, DNA repair and cellular differentiation, playing crucial roles in neuroprotection and neuronal differentiation in neurodegenerative diseases [26]. Conversely, upregulation of mitochondrial and ER antioxidant systems because of reactive oxygen species generation in the brain under oxidative stress conditions illustrated the alteration in lncRNAs on WS treatment in neurodegenerative disorders. Additionally, WS treatment affected neuroinflammatory pathways, particularly those regulated by GPCRs. Neuroinflammation is a well-established contributor to the advancement of neurodegenerative diseases in Alzheimer’s and Parkinson’s, where chronic activation of glial cells leads to the release of pro-inflammatory cytokines as well as neuronal damage [27]. The alterations in lncRNAs involved in GPCR signalling pathways suggested that WS may dampen neuroinflammatory responses, providing a protective effect against inflammatory-mediated neurodegeneration. The modulation in cellular signaling pathways in response to WS treatment in SK-N-SH cells, as observed in the present study, suggested an essential role of lncRNAs in mediating the neuroprotective potential of WS.

Given our historical understanding of the potent therapeutic effects of WS, we tested two dose levels in our study. Interestingly, the higher dose group did not show major differences with the lower dose group in terms of modulation of the lncRNA regulome. This may suggest that lower doses of WS may suffice to elicit its health-beneficial effects. We also studied the temporal effects of exposure of cells to WS at early and late time points that could successfully capture the lncRNA expression. Interestingly, our study showed dramatic differences between the transcriptional response at early and late time points in response to WS. This may be due to time-dependent regulation of intracellular signaling and the complexities of the lncRNA-mRNA co-expression networks. Physiologically, this may translate to altered pharmacokinetics of WS in humans and inter-individual variability. Nonetheless, our results demonstrate that WS may modulate the lncRNA regulome in a time-dependent manner. Further studies are warranted to elucidate the complex mechanisms underlying such temporal cellular events.

The lncRNA BACE1-AS is potential biomarker in AD and upregulated in the brain during AD. In our study, BACE1-AS was significantly downregulated, probably suppressing the formation of disease-related amyloid-beta (Aβ) peptides which are generated from proteolytic cleavage of APP by BACE1, regulated by BACE1-AS [28]. Another lncRNA, MALAT1 was also downregulated and has been known for protection against Aβ_1−42_-induced toxicity. The protective effect of MALAT1 may be due to sponging several miRNAs which further target receptor tyrosine kinase, EPHA2 as well as downstream effector, thus providing protection against Aβ_1−42_-induced cytotoxicity [29].

lncRNAs viz. SNHG1, HOTAIR and MALAT1 mediate dopaminergic neuronal survival along with neuroinflammation in PD. Under PD conditions, SNHG1 is upregulated which acts as a sponge for various miRNAs, thereby affecting various signaling pathways including NLRP3, MAPK1, mTOR and CXCL12. Our findings demonstrated the downregulation of SNHG1 which modulates the expression of genes linked with oxidative stress, autophagy as well as neuroinflammation pathways [30]. Zhang et al. [22]. revealed that the incubation of SH-SY5Y cells with Aβ in an AD cell model resulted in upregulation of lncRNAs SNHG1, RN7S1, SCARNA9 along with downregulation of SNHG16, RGS5, AGAP2-AS and LINC01963. The above findings clearly indicate that modulation in expression of lncRNAs might be linked with Aβ-mediated modifications in AD cell models. Generally, MALAT1 binds to α-Synuclein (α-Syn), enhancing its stability along with enhanced expression of α-Syn [31]. MALAT1 overexpression was downregulated in WS treated cells in our study depicting its neuroprotective effect.

lncRNAs play a significant role in modulation of immune responses, myelin repair processes and neuroinflammation in multiple sclerosis (MS). NEAT1 and KCNQ1OT1 mediate epigenetic regulations affecting immune function as well as inflammatory responses and these biomarkers are upregulated in MS patients. Upregulated NEAT1 and KCNQ1OT1 accounts for TH17/Treg imbalance *via* enhancing T17 cell differentiation and suppression of Treg function [32]. Additionally, lncRNA GAS5 was detected downregulated in our study; it influences activation of T cells and microglial polarization along with oligodendrocyte differentiation [33].

lncRNAs have emerged as key players in epigenetic remodelling, mediating the chromatin landscape along with transcriptional programs governing glioma genesis. Upregulated lncRNA MEG3 exerts tumor-suppressive effects *via* suppression of cell proliferation, induction of apoptosis, learning and memory [34, 35]. In our study, lncRNA MEG3 was upregulated in WS-treated cells, however SNHG1 was downregulated. Evidence on malignant brain tumour tissues has demonstrated the enhanced expression of SNHG1 and is thus associated with malignant progression and unfavourable prognosis of glioma [36].

Antisense SHANK2-AS1 was downregulated in the current study and SHANK2-AS is an important lncRNA in neurodevelopmental psychiatric disorders. SHANK2-AS forms a dsRNA with SHANK2 resulting in inhibition of its expression, effecting neuron structure as well as growth. Previous reports on SHANK2 mutants in mice showed autism spectrum disorder (ASD)-like behaviour depicting the role of SHANK2-AS in synaptic function as well as ASD [37]. Correspondingly, lncRNA BDNF-AS, a naturally present antisense RNA contributing to BDNF expression, has crucial functions in the occurrence of neurodevelopmental disorders [38], and was significantly upregulated in our study. In the co-expression network, it was found that several lncRNAs targeted multiple genes. In this study, important genes such as HMOX1, SLC7A11, HSPA1A, and HSPA1B showed positive correlation with lncRNA expression, whereas CHGB, NEUROG2, NOS1, CHGB, NPY4R2, PROKR2 and INSM2 showed negative correlation with lncRNA expression in WS-treated SK-N-SH cells. It follows that all these identified genes play crucial roles in the gene-noncoding RNA regulation network in neurodegenerative disorders [39–41]. Further, ATP-binding cassette KCNJ which is important in transport of molecules across cell membranes showed negative correlation on WS-treatment underscoring the neuroprotective role of WS in PD [42].

As far as we know, this is the first study comprehensively exploring the lncRNA expression profile of WS-treated SK-N-SH human neuroblastoma cells using RNA sequencing. However, this study still has a few limitations. Firstly, the study was limited to an *in vitro* model i.e., human neuroblastoma cell line. Secondly, out of several differentially expressed lncRNAs, only lncRNAs related to neurodegenerative diseases were discussed. Lastly, the exact mechanism of action of lncRNAs in SK-N-SH cells has not been explored yet and will be the subject of a future study.

## Conclusions

The results generated in this study provide a basis to infer the presence of a lncRNA regulome in neurodegenerative diseases. This regulome includes members of the lncRNA family as well as crosstalk with mRNAs that are co-expressed with lncRNAs. To the best of our knowledge, these regulatory pathways eliciting the health-beneficial effects of WS have not been elucidated till date. Although these outcomes provide valuable comprehensions into the molecular effects of WS, further research is required to validate the specific roles of the modulated lncRNAs. Functional studies, particularly in primary neurons or in vivo models of disease pathogenesis, are essential to confirm the putative roles of these regulatory lncRNAs as novel markers for neurodegenerative diseases which may be amenable for therapeutic intervention.

## Supplementary Information

Below is the link to the electronic supplementary material.

Supplementary material 1 (TIFF 194062.7 kb)

Supplementary material 2 (TIFF 151611.5 kb)

Supplementary material 3 (TIFF 121289.3 kb)

Supplementary material 4 (TIFF 151611.5 kb)

Supplementary material 5 (TIFF 242578.3 kb)

Supplementary material 6 (TIFF 87011.9 kb)

Supplementary material 7 (TIFF 151611.5 kb)

Supplementary material 8 (XLSX 2779.4 kb)

Supplementary material 9 (XLSX 2616.2 kb)

Supplementary material 10 (XLSX 2968.7 kb)

Supplementary material 11 (XLSX 2664.9 kb)

Supplementary material 12 (XLSX 2260.1 kb)

Supplementary material 13 (XLSX 2404.6 kb)

Supplementary material 14 (XLSX 2414.4 kb)

Supplementary material 15 (XLSX 2504.5 kb)

## Data Availability

The datasets presented in this study can be found in online repositories. The names of the repository/repositories and accession number(s) can be found below: [https://www.ncbi.nlm.nih.gov/](https:/www.ncbi.nlm.nih.gov), SRA repository, BioProject accession number PRJNA1168412.
